# Diagnostic Classification of Bruch's Membrane Opening-Minimum Rim Width and Retinal Nerve Fiber Layer Thickness in Myopic Eyes by Optical Coherence Tomography

**DOI:** 10.3389/fmed.2021.729523

**Published:** 2021-08-25

**Authors:** Geng Wang, Miaoru Zhen, Shasha Liu, Kunliang Qiu, Cui Liu, Ji Wang, Mingzhi Zhang

**Affiliations:** Joint Shantou International Eye Center of Shantou University and The Chinese University of Hong Kong, Shantou, China

**Keywords:** myopia, optical coherence tomography, Bruch's membrane opening-minimum rim width, retinal nerve fiber layer, diagnostic classification

## Abstract

**Purpose:** This study was conducted in order to compare the diagnostic classification of Bruch's membrane opening-minimum rim width (BMO-MRW) and RNFL thickness in normal myopic subjects by using optical coherence tomography (OCT).

**Methods:** This cross-sectional study involved 75 healthy myopic subjects [spherical equivalent (SE) ≤ −0.5D] from April 2019 to January 2020. One eye of each subject was randomly selected for examination. BMO-MRW and peripapillary RNFL thickness were measured by spectral-domain OCT (Spectralis, Heidelberg Engineering GmbH, Heidelberg, Germany). All the subjects were divided into three groups: low myopic group (SE > −3D), moderate myopic group (−6D < SE ≤ −3D), and high myopic group (SE ≤ −6D). A nonparametric test was used to analyze the difference among groups. Linear regression was used to analyze the relationship between BMO-MRW/RNFL thickness and axial length/spherical equivalent. McNemar test was used to compare the diagnostic classification between BMO-MRW and RNFL thickness.

**Results:** The RNFL thickness classified a significantly higher percentage of eyes as outside normal limits/borderline in at least 1 quadrant (BMO-MRW, 4%; RNFL thickness, 34.67%; *p* < 0.01). There was no significant correlation between BMO-MRW/RNFL thickness and AL/SE. The low myopia (SE > −3D) had a significantly lower percentage of eyes classified as outside normal limits/borderline in at least 1 quadrant than the moderate myopia (−6D < SE ≤ −3D) and high myopia (SE ≤ −6D) (low myopia, 12.5%; moderate/high myopia, 42.42%/50%; *p* < 0.05).

**Conclusion:** BMO-MRW had a lower percentage of eyes classified as outside normal limits/borderline in at least 1 quadrant than RNFL thickness in normal myopic subjects. When referring to the diagnostic classification of RNFL thickness in myopic subjects, caution should be exercised in interpreting positive results. Further studies are needed to compare the diagnostic accuracy of these two measurements in myopic glaucoma patients.

## Introduction

Myopia is a common ocular disorder worldwide. It is estimated that there will be 4,758 million people with myopia in 2050 (1, 2). Myopia is a risk factor for primary open-angle glaucoma (POAG) that can increase the risk of POAG by two- to three-fold (3, 4). Since POAG can cause asymptomatic vision loss, early diagnosis of POAG in myopic patient is important. Diagnosis of POAG relies on evaluating optic disc, visual field, intraocular pressure (IOP), retinal nerve fiber layer (RNFL), and retinal ganglion cell (RGC) layer. However, myopic eye is associated with tilted optic disc (5, 6), thinning of the RNFL (7) and RGC layer (8), increased intraocular pressure (4), and visual field defects (9). As a result, the diagnosis of POAG in myopic patients is challenging.

RNFL thickness measured by optical coherence tomography (OCT) is widely used for early diagnosis of POAG. It was reported that RNFL thickness measurement would be affected by myopia (7, 10). A significant proportion of normal myopic eyes were reported to be classified as abnormal (outside normal limits or borderline) (11, 12). Bruch's membrane opening-minimum rim width (BMO-MRW) represents the least distance between the Bruch's membrane opening and the internal limiting membrane, through which axons of RGCs pass. It has been shown that measuring BMO-MRW may have a higher accuracy in detecting glaucoma (13). To our knowledge, no study has been performed to compare the diagnostic classification of RNFL thickness and BMO-MRW in normal myopic subjects. The aim of this study was to compare the diagnostic classification of RNFL thickness and BMO-MRW in normal myopic subjects with spectral domain OCT.

## Methods

This was a cross-sectional study. The study was designed following the ethical standards of the Declaration of Helsinki and approved by the ethical committees of the Joint Shantou International Eye Center of Shantou University and The Chinese University of Hong Kong with written informed consent obtained before the study.

### Subjects

Eighty-seven Chinese subjects with spherical equivalent (SE) ranging from −0.5 to −10.75D were recruited in the current study. One eye from each subject was randomly selected. All subjects received a comprehensive ocular examination, including best corrected visual acuity, intraocular pressure (IOP), slit lamp examination, refraction, axial length (AL) (OA-2000, Tomey, Japan) and 24-2 standard automated perimetry.

### Inclusion and Exclusion Criteria

All the included eyes have SE of less than −0.5D and no other concurrent diseases. Subjects with best corrected visual acuity of less than 20/40, IOP over 21 mmHg, family history of glaucoma, intraocular surgery, myopic macular degeneration, clinical evidence of glaucoma, parapapillary atrophy extending the circle of OCT RNFL scan, refractive surgery, age less than 18, neurological diseases, and diabetes and subjects unable to cooperate with the examination or with poor OCT image quality were excluded.

### Visual Field Testing

All visual field tests were performed with the static automated white-on-white threshold 24-2 SITA standard strategy (Humphrey Field Analyzer II; Carl Zeiss Meditec, Inc., Dublin, CA, USA). A visual field test was considered to be reliable when false-positive errors were less than 15%, false-negative errors were less than 15%, and fixation losses were less than 20%. All the visual field tests of the included eyes were “within normal limits” in the glaucoma hemifield test (GHT).

### Spectralis OCT Imaging

Imaging of Spectralis OCT (Spectralis, Heidelberg Engineering GmbH, Heidelberg, Germany; Spectralis family acquisition module, version 6.0.11.0) was conducted using the Glaucoma Module Premium Edition (GMPE; Heidelberg Engineering). Radial B-scans of 24 were acquired for BMO-MRW. The OCT images were 15° in width and were obtained at 7.5° intervals of the ONH. Three scan circles (3.5, 4.1, and 4.7 mm in diameter) were used to measure peripapillary RNFL thickness. Well-centered scans with accurate retinal segmentation and image quality higher than 20 were included. The axis between the BMO center and fovea (fovea–BMO axis, FoBMO axis) was attained before data collection and analyses. Data collection and analyses of six quadrants (N, nasal; NS, superonasal; NI, inferonasal; T, temporal; TS, superotemporal; TI, inferotemporal) were achieved with regard to FoBMO axis. The diagnostic classifications presented with three colors were attained by comparing with the imbedded normative database. Green represents over 95% normal results and was considered as within normal limits. Yellow represents 1~5% normal results and was considered as borderline. Red represents less than 1% normal results and was considered as outside normal limits.

### Statistical Analyses

Statistical analyses were performed with SPSS version 21.0 (SPSS, Chicago, IL, USA). The data were presented as mean ± standard deviation. Normal distribution was tested by the Shapiro–Wilk test. Linear correlation analysis was used to investigate the correlation between BMO-MRW/RNFL and SE/AL. McNemar test was used to compare the diagnostic classification between BMO-MRW and RNFL thickness. A *p*-value < 0.05 was considered statistically significant.

## Results

Eighty-seven eyes from 87 Chinese subjects were involved in this study. Twelve eyes were excluded for unacceptable visual field tests or OCT scans. Finally, 75 eyes from 75 Chinese subjects (54 females and 37 right eyes) were included in the current study. Mean age, SE, and axial length were 31.08 ± 10.32 years (range: 20 to 64), −4.13 ± 2.29D (range: −10.75 to −0.50D), and 25.07 ± 1.19 mm (range: 22.22 to 27.55), respectively. All the subjects were divided into three groups: low myopic group (SE > −3D), moderate myopic group (−6D < SE ≤ −3D), and high myopic group (SE ≤ −6D). There was no significant difference in age, gender, and visual field average loss between three groups. Their baseline information is presented in Table 1.

**Table 1:** Characteristics of the high, moderate, and low myopia groups.

**Characteristics (mean ± SD)**	**Low myopia (*n* = 24)**	**Moderate myopia (*n* = 33)**	**High myopia (*n* = 18)**	***p*-value**
Age (years)^a^	30.50 ± 9.43	33.45 ± 12.16	27.50 ± 6.33	0.12
Gender, male/female^b^	3/21	12/21	6/12	0.12
Spherical equivalent (D)^a^	−1.61 ± 0.57	−4.26 ± 0.86	−7.25 ± 1.32	<0.01
Axial length (mm)^a^	24.03 ± 0.88	25.25 ± 0.90	26.11 ± 0.93	<0.01
MD (3)^c^	−1.35 ± 1.36	−1.20 ± 1.39	−1.72 ± 1.03	0.39
PSD (3)^a^	1.36 ± 0.29	1.51 ± 0.48	1.52 ± 0.27	0.11

*Low myopia: SE > −3D; moderate myopia: −6D < SE ≤ −3D; high myopia group: SE ≤ −6D. MD, mean deviation of visual field; PSD, pattern SD of visual field; SD, standard deviation*.

a *Kruskal–Wallis test*.

b *Chi-square test*.

c *One-way ANOVA*.

### BMO-MRW and RNFL Measurement

The mean BMO-MRW was 369.75 ± 61.05 μm. There was no significant correlation between BMO-MRW and AL/SE. No significant correlation was found between BMO-MRW of each quadrant and AL/SE. No significant difference of BMO-MRW was found among the three groups (Table 2).

**Table 2:** Correlations between Bruch's membrane opening-minimum rim width and axial length/spherical equivalent.

	**All groups (*n* = 75)**	**Low myopic group (*n* = 24)**	**Middle myopic group (*n* = 33)**	**High myopic group (*n* = 18)**	***p*-value^**a**^**	**Axial length**		**Spherical equivalent**	
						***r***	***p*-value**	***r***	***p*-value**
Global	369.75 ± 61.05	368.67 ± 63.64	367.76 ± 57.72	374.83 ± 66.59	0.99	0.20	0.08^b^	−0.07	0.54^c^
Temporal	265.93 ± 55.50	269.13 ± 56.37	259.03 ± 55.98	274.33 ± 55.02	0.52	0.22	0.05^c^	−0.07	0.54^c^
Superotemporal	367.31 ± 73.80	365.96 ± 68.26	362.06 ± 75.06	378.72 ± 81.28	0.84	0.19	0.10^b^	−0.08	0.50^c^
Inferotemporal	396.67 ± 73.94	407.79 ± 68.68	384.46 ± 71.55	404.22 ± 85.18	0.44	0.17	0.15^b^	−0.03	0.78^c^
Nasal	404.20 ± 71.93	395.88 ± 80.83	410.27 ± 64.42	404.17 ± 75.49	0.69	0.17	0.14^b^	−0.06	0.62^c^
Superonasal	423.49 ± 74.61	421.08 ± 69.92	425.70 ± 74.05	422.67 ± 85.30	0.93	0.19	0.10^b^	−0.06	0.60^c^
Inferonasal	430.25 ± 70.91	429.63 ± 68.81	425.94 ± 66.01	439.00 ± 84.62	0.84	0.14	0.24^b^	−0.11	0.33^c^

*BMO-MRW, Bruch's membrane opening-minimum rim width (μm)*.

a *Kruskal–Wallis test*.

b *Pearson correlation analysis*.

c *Spearman correlation analysis*.

The mean RNFL thickness was 109.25 ± 9.20 μm. Both the temporal (*r* = 0.33, *p* < 0.01) and superotemporal (*r* = 0.32, *p* < 0.01) RNFL thickness were positively correlated with axial length. The inferonasal (*r* = −0.32, *p* < 0.01) RNFL thickness was negatively correlated with axial length. No significant correlation was found between RNFL thickness of other quadrants and AL/SE. No significant difference of RNFL thickness was found among the three groups (Table 3).

**Table 3:** Correlations between RNFL thickness and axial length/spherical equivalent.

	**All groups (*n* = 75)**	**Low myopic group (*n* = 24)**	**Middle myopic group (*n* = 33)**	**High myopic group (*n* = 18)**	***p*-value^**a**^**	**Axial length**		**Spherical equivalent**	
						***r***	***p*-value**	***r***	***p*-value**
Global	109.25 ± 9.20	111.42 ± 7.52	106.67 ± 9.87	111.11 ± 9.22	0.21	0.09	0.44^b^	0.05	0.70^c^
Temporal	94.49 ± 22.48	90.63 ± 14.51	93.76 ± 25.77	101.00 ± 24.44	0.30	0.33	<0.01^c^	−0.22	0.06^c^
Superotemporal	153.05 ± 23.45	155.29 ± 19.06	147.79 ± 25.69	159.72 ± 23.52	0.28	0.32	<0.01^b^	−0.08	0.52^c^
Inferotemporal	173.44 ± 24.41	178.33 ± 17.29	169.00 ± 21.72	175.06 ± 35.03	0.19	−0.10	0.39^b^	0.14	0.24^c^
Nasal	73.43 ± 14.29	76.42 ± 8.53	71.70 ± 16.63	72.61 ± 15.85	0.40	−0.11	0.37^b^	0.21	0.08^c^
Superonasal	131.19 ± 29.24	138.13 ± 29.61	126.42 ± 30.67	130.67 ± 25.55	0.41	−0.09	0.46^c^	0.15	0.19^c^
inferonasal	111.97 ± 23.72	118.00 ± 23.99	109.91 ± 25.33	107.72 ± 19.57	0.43	−0.32	<0.01^b^	0.15	0.20^c^

*RNFL, retinal nerve fiber layer (μm)*.

a *Kruskal–Wallis test*.

b *Pearson correlation analysis*.

c *Spearman correlation analysis*.

### BMO-MRW and RNFL Diagnostic Classification Result

All the global BMO-MRW were classified as normal. For the temporal quadrant, 2 of 75 eyes (2.67%) were classified as abnormal. For the superotemporal and inferotemporal quadrant, 1 of 75 eyes (1.37%) was classified as borderline.

For the global RNFL thickness, 73 eyes (97.33%) were classified as normal, while 2 eyes (2.67%) were classified as borderline. For the nasal quadrant, 19 eyes (25.33%) were classified as abnormal (borderline, 6; outside normal limits, 13). All RNFL thickness was classified as normal for the temporal quadrant. Table 4 presents the proportion of eyes identified as abnormal based on the normative databases for BMO-MRW and RNFL thickness.

**Table 4:** Diagnostic classification results of BMO-MRW and RNFL (*n* = 75).

	**BMO-MRW**			**RNFL**		
	**Within normal limits**	**Borderline**	**Outside normal limits**	**Within normal limits**	**Borderline**	**Outside normal limits**
Global	75 (100%)	0	0	73 (97.33%)	2 (2.67%)	0
Temporal	73 (97.33%)	2 (2.67%)	0	75 (100%)	0	0
Superotemporal	74 (98.67%)	1 (1.33%)	0	73 (97.33%)	2 (2.67%)	0
Inferotemporal	74 (98.67%)	1 (1.33%)	0	73 (97.33%)	0	2 (2.67%)
Nasal	75 (100%)	0	0	56 (74.67%)	6 (8.00%)	13 (17.33%)
Superonasal	75 (100%)	0	0	72 (96.00%)	2 (2.67%)	1 (1.33%)
Inferonasal	75 (100%)	0	0	67 (89.33%)	6 (8.00%)	2 (2.67%)

*BMO-MRW, Bruch's membrane opening-minimum rim width; RNFL, retinal nerve fiber layer*.

Abnormal classification was considered as false positive and was defined as “borderline” or outside normal limits in the current study. For quadrants, the RNFL thickness had a significantly higher percentage of eyes detected as abnormal (RNFL thickness, 34.67%; BMO-MRW, 4.00%; *p* < 0.01, McNemar test).

In the diagnostic classification results of BMO-MRW, the low myopic group had one false-positive result (4.17%), the moderate myopic group had two false-positive results (6.06%), and the high myopic group had no abnormal result. However, there was no significant difference among the three groups (χ^2^ = 1.12, *p* = 0.79).

In the diagnostic classification results of RNFL, the low myopic group had 3 false-positive results (12.50%), the moderate myopic group had 14 false-positive results (42.42%), and the high myopic group had 9 false-positive results (50.00%). There was a significant difference between the three groups (χ^2^ = 7.95, *p* = 0.02). The low myopic group had a significant difference with the moderate/high myopic groups (*p* < 0.05), while the moderate myopic group had no significant difference with the high myopic group.

## Discussion

This study found that there was no significant correlation between BMO-MRW/RNFL thickness and AL/SE. However, both the temporal and superotemporal RNFL thickness were positively correlated with AL. The false-positive rate of BMO-MRW diagnostic classification was significantly lower than RNFL thickness.

Previous studies found that RNFL became thinner with increasing myopia (11). However, Kang et al. (14) found that mean RNFL thickness decreased with increasing myopia, and after the ocular magnification adjustment, the mean RNFL thickness had no correlation with myopia. In this study, we used different versions of OCT which adjusted the ocular magnification during the measurement of RNFL. The result showed that mean RNFL had no significant correlation with axial length and SE in this study. Zheng et al. (15) found that superotemporal and inferotemporal RNFL yield the best diagnostic performance for glaucoma. In this study, superotemporal RNFL thickness had a significant correlation with axial length. So the influence of axial length on superotemporal RNFL thickness may affect the diagnosis of glaucoma with myopia. Due to this, it is necessary to build a normative database of RNFL thickness for myopia.

It has been reported that BMO-MRW has a higher sensitivity and specificity in diagnosing glaucoma (16, 17). BMO-MRW also has a better correlation with glaucomatous visual field defect (18). BMO is an anatomical mark of the outer edge of the optic disc. The measurement of BMO-MRW is based on the edge of the optic disc which would not be affected by the distance from the optic disc. As a result, BMO-MRW might not be affected by the ocular magnification due to the axial length extension. Sastre-Ibañez et al. (19) reported that BMO-MRW had no significant difference between moderate myopic subjects and low/nonmyopic subjects. In this study, axial length and SE were not correlated with BMO-MRW. BMO-MRW had no significant difference among the three groups. BMO-MRW measurement was not affected by axial myopia. Therefore, BMO-MRW measurements might be more suitable for myopic subjects when screening glaucoma.

Nowadays, the diagnostic classification result plays an important role in diagnosing glaucoma. OCT can provide the diagnostic classification result after comparing the BMO-MRW and RNFL measurements with its normal database. For RNFL thickness, Leung et al. found that there were up to 45.2% healthy myopic subjects diagnosed as abnormal. Qiu et al. (12) found that there were up to 55.7% myopic subjects diagnosed as abnormal by Cirrus OCT, and the abnormal results mainly occurred in the nasal sector of the optic disc. In this study, the false-positive rate was 34.67% and the nasal sector had the highest false-positive rate. Kang et al. (14) pointed out that myopia may be associated with temporal deviations in the peak RNFL thickness and this may cause temporal RNFL thickening in myopia. Therefore, nasal RNFL might have a higher false-positive rate due to RNFL thinning in the nasal sector. In addition, both the moderate myopic group and the high myopic group had higher false-positive rates than the low myopic group, and caution should be exercised when analyzing the positive result of the moderate/high myopic subjects. BMO-MRW had a lower false-positive rate than RNFL in moderate myopic subjects (19). In this study, the false-positive rate of BMO-MRW measurement in healthy myopic subjects was 4.00% which was significantly lower than the RNFL result (34.67%). This indicated that BMO-MRW measurement reduced the false-positive rate caused by myopia.

However, BMO-MRW also had three false-positive results in this study, while the visual fields and intraocular pressures were within the normal range. Two of them had “borderline” results in the temporal sector, while RNFL measurement results were classified as normal. The remaining one was classified as “borderline” in superotemporal and inferotemporal BMO-MRW, while the mean superotemporal and inferonasal RNFL were classified as “borderline.” Therefore, early glaucomatous damage cannot be excluded and follow-up is necessary.

In this study, the temporal sector of BMO-MRW had the highest false-positive rate, while the highest false-positive rate of RNFL occurred in the nasal sector which was in the opposite position of BMO-MRW. With the development of axial myopia, the elongation of the globe takes place more in the posterior segment so that the position of the optic nerve moves relatively to the nasal side of the posterior segment. However, the Bruch's membrane opening would not shift with the optic nerve (20). It leads to the absence of the Bruch's membrane at the temporal disc border which can explain the high false-positive rate of BMO-MRW. On the other hand, Bruch's membrane defects can lead to the corresponding retinal pigment epithelium and choroid defects, so the temporal atrophy of the optic disc should be taken into account for the abnormal results of temporal BMO-MRW. The peak of RNFL thickness had a trend of temporal deviation with increasing myopia which can explain the high false-positive rate of the nasal RNFL diagnostic classification results. However, nasal RNFL thickness had no significant correlation with axial length and SE. It is known that supero- and inferotemporal RNFL sectors are the most sensitive parameters for diagnosing glaucoma. In the current study, the positive rates of RNFL and BMO-MRW were similar in supero- and inferotemporal sectors (1.33 vs. 2.67% for both sectors). The highest positive rate occurred in the nasal sector of RNFL. Since the abnormal nasal sector of RNFL may not affect the diagnosis of glaucoma, longitudinal studies are needed to address this issue.

During the examination of OCT, it is very important to confirm the exact position of BMO so that we can obtain a reliable BMO-MRW value. Zheng et al. (21) found that high myopic eyes were more likely to have indiscernible BMO at the temporal, superotemporal, and inferotemporal sectors of the optic disc. Increased axial length, parapapillary atrophy, and advanced glaucoma affect the location of BMO. These may compromise the measurement of neuroretinal rim in the diagnostic evaluation of glaucoma. Therefore, it is necessary to confirm the position of BMO before further analyses when using BMO-MRW as the detection parameter of glaucoma. In addition, the resolution of OCT and the analytical ability to recognize BMO of the computer software need to be improved.

This study has some limitations. In this study, about half of the subjects were in the moderate myopic group which may result in an uneven distribution of sample size. The statistical methods were used to reduce bias. During the study process, 12 subjects were excluded because of abnormal visual fields (11 “borderline” and 1 “general sensitivity decline”). Since myopia can be related to visual field defect, the otherwise normal myopic subjects may be excluded. On the other hand, we did not measure other parameters of optic disc, so further study about myopic optic disc morphology affecting RNFL and BMO-MRW is needed. In addition, although BMO-MRW reduced the false-positive results of myopic subjects, the advance of glaucoma may cause other changes in ocular structure. Furthermore, the diagnostic accuracy of both measurements in myopic glaucoma was not evaluated in the current study. Therefore, comparison with glaucomatous subjects is needed to make the study more complete.

In summary, BMO-MRW had a lower false positive than RNFL thickness in normal myopic subjects. When referring to the diagnostic classification of RNFL thickness in myopic subjects, caution should be exercised in interpreting positive results. However, the accuracy of both measurements in myopic glaucoma patients was not evaluated in the current study. Further studies are needed to compare the diagnostic accuracy of these two measurements in myopic glaucoma patients.

## Data Availability Statement

The excel data used to support the findings of this study is available from author Geng Wang upon request. Requests to access these datasets should be directed to Geng Wang, wg@jsiec.org.

## Ethics Statement

The studies involving human participants were reviewed and approved by ethical committee of Joint Shantou International Eye Center of Shantou University and The Chinese University of Hong Kong. The patients/participants provided their written informed consent to participate in this study.

## Author Contributions

All authors listed have made substantial, direct and intellectual contribution to the work and approved it for publication.

## Conflict of Interest

The authors declare that the research was conducted in the absence of any commercial or financial relationships that could be construed as a potential conflict of interest.

## Publisher's Note

All claims expressed in this article are solely those of the authors and do not necessarily represent those of their affiliated organizations, or those of the publisher, the editors and the reviewers. Any product that may be evaluated in this article, or claim that may be made by its manufacturer, is not guaranteed or endorsed by the publisher.
